# Classification of Alzheimer's Disease, Mild Cognitive Impairment, and Normal Controls With Subnetwork Selection and Graph Kernel Principal Component Analysis Based on Minimum Spanning Tree Brain Functional Network

**DOI:** 10.3389/fncom.2018.00031

**Published:** 2018-05-09

**Authors:** Xiaohong Cui, Jie Xiang, Hao Guo, Guimei Yin, Huijun Zhang, Fangpeng Lan, Junjie Chen

**Affiliations:** ^1^College of Information and Computer, Taiyuan University of Technology, Taiyuan, China; ^2^Department of Computer Science and Technology, Taiyuan Normal University, Taiyuan, China; ^3^Department of Digital Media Technology, Communication University of Shanxi, Jinzhong, China

**Keywords:** minimum spanning tree, gSpan, graph kernel principal component analysis, mild cognitive impairment, Alzheimer's disease, classification

## Abstract

Effective and accurate diagnosis of Alzheimer's disease (AD), as well as its early stage (mild cognitive impairment, MCI), has attracted more and more attention recently. Researchers have constructed threshold brain function networks and extracted various features for the classification of brain diseases. However, in the construction of the brain function network, the selection of threshold is very important, and the unreasonable setting will seriously affect the final classification results. To address this issue, in this paper, we propose a minimum spanning tree (MST) classification framework to identify Alzheimer's disease (AD), MCI, and normal controls (NCs). The proposed method mainly uses the MST method, graph-based Substructure Pattern mining (gSpan), and graph kernel Principal Component Analysis (graph kernel PCA). Specifically, MST is used to construct the brain functional connectivity network; gSpan, to extract features; and subnetwork selection and graph kernel PCA, to select features. Finally, the support vector machine is used to perform classification. We evaluate our method on MST brain functional networks of 21 AD, 25 MCI, and 22 NC subjects. The experimental results show that our proposed method achieves classification accuracy of 98.3, 91.3, and 77.3%, for MCI vs. NC, AD vs. NC, and AD vs. MCI, respectively. The results show our proposed method can achieve significantly improved classification performance compared to other state-of-the-art methods.

## Introduction

Alzheimer's disease (AD) is a common and progressive neurodegenerative disorder of the nervous system. It is predicted that the number of AD patients will double in the next 20 years (Brookmeyer et al., 2007). Therefore, in its early stage, such as mild cognitive impairment (MCI), early diagnosis and treatment for patients is of great significance for delaying the development of the disease. However, because of the subtle differences between AD, MCI and Normal Control (NC) in cognitive function, MCI is more difficult to diagnose. Therefore, it is very crucial to propose methods that can identify diagnostic markers of MCI and AD, and classify AD, MCI, and NC.

Over the past 10 years, technologies such as functional magnetic resonance imaging (fMRI) and electroencephalograph (EEG) have emerged, which provide effective and non-invasive ways to capture human brain's functional connectivity patterns. Recently, neuroimaging techniques, such as structural magnetic resonance imaging (sMRI) (Aguilar et al., 2013; Li et al., 2014; Beheshti et al., 2015; Moradi et al., 2015; Papakostas et al., 2015), functional MRI (fMRI) (Andersen et al., 2012; Dinesh et al., 2013), Diffusion Tensor Imaging (DTI) (Graña et al., 2011; Mesrob et al., 2012; Lee et al., 2013), Positron Emission Tomography (PET), and Single Photon Emission Computed Tomography (SPECT) (Hanyu et al., 2010; Górriz et al., 2011; Gray et al., 2012; Chen et al., 2013) have been used successfully in the classification of AD and MCI.

Supekar et al. (2008) used the clustering coefficient as feature to identify AD from normal controls with specificity of 78% and sensitivity of 72%. Zhang et al. (2011) proposed a multimodal [MRI, PET, and Cerebrospinal Fluid (CSF)] classification framework to discriminate between AD and normal controls by using a kernel combination method with accuracy of 93.2%. Wee et al. (2012b) integrated anatomical and functional connectivity information to identify MCI from normal controls by using a multiple-kernel-based support vector machine algorithm with accuracy of 96.3%. Wee et al. (2012a) extracted clustering coefficient of five frequency subbands for classification. The classification accuracy was 86.5%. Jie et al. (2014a) integrated multiple properties of a connectivity network for identifying MCI with accuracy of 91.9%. Jie et al. (2014b) proposed a classification framework to identify MCI by using a set of local measures and topological information derived from functional connectivity networks. This method achieved area under receiver operating characteristic curve of 0.94, classification accuracy of 91.9%, and sensitivity of 100.0%.

A common problem in the above studies was to use network properties based on threshold connected network for AD and MCI classification. However, this may affect the final classification performance to some extent, because to construct threshold function network, we need to set a threshold for the original weighted network. Threshold can be set according to connectivity strength or network density. When threshold is set based on connectivity strength, due to the difference of the connectivity weight distribution of the original network, two different density networks are generated, and these differences affect the network characteristics. When threshold is set based on network density, though the number of connections is fixed, it may cause false or noisy connections in the network or exclude related connections in the network. This may be a good explanation for some contradictory results in the study of brain disease (Diessen et al., 2013). Therefore, in order to solve these problems, researchers often study network attributes in a range of thresholds. Supekar et al. (2008) set threshold from 0.01 to 0.99 to study the small world properties of AD functional connectivity networks. Zanin et al. (2012) studied classification performance within an appropriate threshold range in order to find the best threshold. Geng et al. (2017) studied the graph theory of the brain network in the sparsity threshold from 0.17 to 0.5.

In 2015, Tewarie et al. proposed that the minimum spanning tree (MST) is an unbiased approach to the construction and analysis of brain networks. The construction of MST depends only on the ordering of weights in the original network, and does not depend on the distribution of these weights or the absolute value (Dobrin and Duxbury, 2001; Jackson and Read, 2010). In addition, in many fields of science, it is found that MST can effectively capture the essential attributes of complex networks. In 2006, Lee et al. applied MST to brain network for the first time, and MST was widely applied to the research and development of many kinds of neuropsychiatric disorders (Lee et al., 2006; Boersma et al., 2012; Demuru et al., 2013; Stam et al., 2014; Guo et al., 2017a,b).

Accordingly, in this article, we propose a classification framework to accurately identify multiclass (MCI patients, AD patients, and NCs) by using topological information derived from MST brain networks. Our approach uses three new tools: Kruskal's algorithm (Kruskal, 1956), gSpan (Yan and Han, 2002), and graph kernel PCA (Schölkopf et al., 1997). Specifically, Kruskal's algorithm was used to construct the brain functional network, and gSpan was used to extract features. Moreover, graph kernel PCA was used to select features by directly measuring the topological similarity between brain networks.

**Figure 1:**
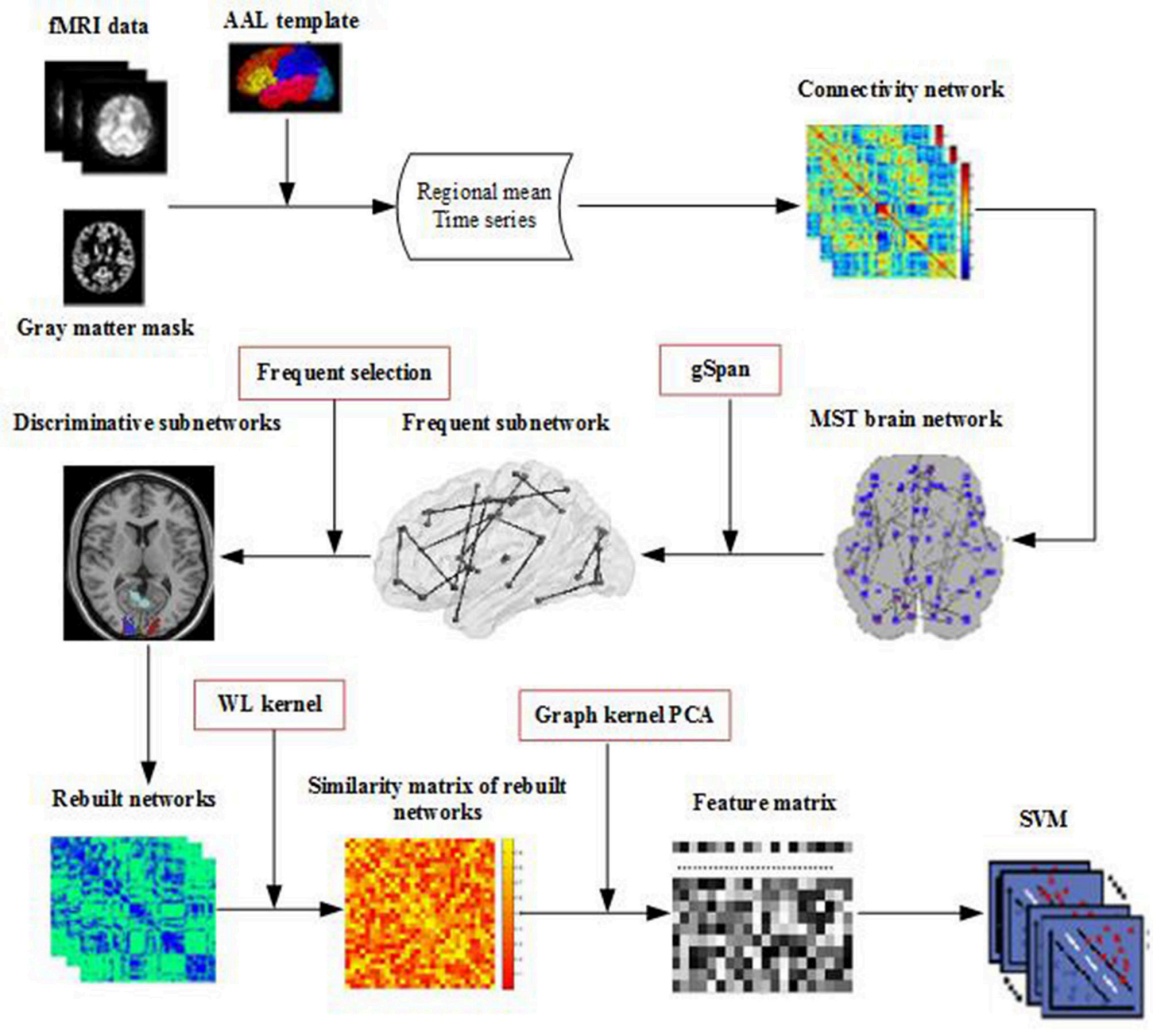
Framework of proposed method.

Figure 1 illustrates the framework of our proposed method. Specifically, for each subject, a brain network is constructed firstly by MST from the respective fMRI data. Then, frequent subnetworks are mined by gSpan from the respective MST, and the most discriminative subnetworks are selected using the subnetwork selection algorithm based on their respective frequency differences. Moreover, graph kernel PCA is used to extract features from the rebuilt networks. Finally, a Support Vector Machine (SVM) is used to classify the data with extracted features.

## Materials and methods

### Data acquisition and preprocessing

The data used in this study was downloaded from the Alzheimer's Disease Neuroimaging Initiative (ADNI) database (http://adni.loni.usc.edu/). ADNI was launched in 2003 by the National Institute on Aging (NIA). It aims to study the pathogenesis of AD by analyzing a variety of medical imaging data.

This study included a total of 68 participants, including 21 AD patients, 25 MCI patients, and 22 NCs. Table 1 shows the demographic information of the participants. Details of the acquisition process and scanning parameters are available on the ADNI website (http://adni.loni.usc.edu/data-samples/).

**Table 1:** Demographic information of study participants.

**Group**	**AD**	**MCI**	**NC**
No. of subjects (M/F)	8/13	16/9	10/12
Age (mean ±*SD*)	74.1 ± 7.4	73.5 ± 6.1	74.9 ± 6.3
MMSE (mean ±*SD*)	21 ± 3.5	27.6 ± 2.0	28.8 ± 1.7
CDR (mean ±*SD*)	0.8 ± 0.2	0.5 ± 0.2	0.0 ± 0.1

*AD, Alzheimer's disease patients; MCI, Mild cognitive impairment; NC, Normal control; MMSE, Mini-mental state examination; CDR, Clinical dementia rating; M, Male; F, Female*.

Many preprocessing steps of the fMRI images, which includes brain skull removal and motion correction followed by temporal pre-whitening, spatial smoothing, global drift removal, slice time correction, and band pass filtering, were performed using the Statistical Parametric Mapping software package (http://www.fil.ion.ucl.ac.uk/spm/software/spm12/). Then, by warping the Automated Anatomical Labeling (AAL) template (Tzourio-Mazoyer et al., 2002), for each subject, we parcellated the brain space into 90 regions of interest (ROIs). For each individual ROI, the fMRI time series of all voxels was averaged to be the mean time series of the ROI. Lastly, with ROIs as nodes and Pearson correlation coefficients between pair of ROIs as connectivity weight value, a functional full connected network was constructed for each subject.

### Methods

The key techniques of the classification framework included Kruskal's algorithm to construct the MST brain network, gSpan, and graph kernel PCA to select features. Kruskal's algorithm was used to construct the unbiased brain networks. In order to extract the topological properties of networks, gSpan was adopted to mine frequent subnetworks from the brain network, and then the discriminative subnetworks were selected according to the frequency difference. To reflect the differences in the topology of brain networks, graph kernel PCA was used to measure the similarity between the brain networks. Finally, the topological structure features was used to classify.

#### MST network construction

**Definition 1 (Minimum spanning tree)**. For a connected, edge-weighted undirected graph G, an MST of G is a subset of the edges that connect all vertices without any cycles and with the minimum possible total edge weight.

Kruskal's algorithm is a well-known algorithm for finding an MST. In our case, we start the algorithm with the largest link weights because we are interested in the strongest connections in the brain network. This algorithm first orders the weights of all links in descending order, then constructs the MST with the largest link weight, and finally adds the following largest link weights until all nodes are connected in an acyclic subnetwork consisting of links. This link is ignored, if the addition of a link forms a loop.

#### gSpan

Because it is difficult to get the discriminative subnetworks directly, we need to mine the frequent subgraph firstly. We use the gSpan algorithm to mine frequent subnetworks from the MST the brain network. gSpan finds frequent subnetworks by using a depth-first-search method. In this regard, some preliminaries are discussed below.

**Definition 2 (Undirected labeled network)**. For an undirected labeled network G = (V, E, L), V represents the set of nodes; E ⊆ V × V, the set of edges; L, the set of labels.

**Definition 3 (Subnetwork)**. Given two undirected labeled networks G = (V, E, L) and G_s_ = (V_s_,_E_s_, *L*s_), if V_s_ ⊆ V, L_s_ ⊆ L and E_s_ ⊆ E, G_s_ is a subnetwork of G.

**Definition 4 (Subnetwork frequency)**. For a given network set *G* = {*G*_1_, *G*_2_, ⋯*G*_*n*_}, n is the number of networks. The frequency f_q_ of a subnetwork g_s_ is defined as follows:

(1) $${\text{f}}_{\text{q}}\left({\text{g}}_{\text{s}}|𝔾\right)=\frac{\left|{\text{g}}_{\text{s}}\text{ is subgraph of G and G }\in 𝔾\right|}{\left|𝔾\right|}$$

**Definition 5 (Frequent subnetwork mining)**. For a given undirected labeled network set *G* and frequency thresholding value s where 0 ≤ s ≤ 1, the process of finding all subnetworks of *G* with frequency of at least s is called frequent subnetwork mining.

#### Discriminative subnetworks selection

In fact, there are a large number of frequent subnetworks in a network, but only a small portion of the frequent subnetworks have discriminability. Therefore, the most discriminative subnetworks were selected by using the further feature selection method based on their respective frequency differences. The greater the frequency difference, the stronger the discriminability. Then, the brain network is reconstructed using the most discriminative subnetworks. Specifically, for a network, we only need to delete edges that do not appear in any discriminative subnetworks. In this way, the topology of the brain network and the discriminative subnetworks are preserved.

#### Graph kernel PCA

If the discriminative subnetworks are directly used for classification, the topological properties of the brain network will be lost. Therefore, it is necessary to extract the topological properties of the brain network.

In this section, graph kernel PCA is used to extract the topological features from rebuilt brain networks. Based on the rebuilt brain networks, the graph kernel is used to map the brain network from the original network space to the feature space and measure the similarity between two brain networks by comparing their topological structures. In this study, we use the Weisfeiler–Lehman (WL) subtree kernel (Shervashidze et al., 2011) because it can effectively capture topological information and measure similarity of networks (Du et al., 2016).

For a pair of brain networks G and H, the basic process of the WL subtree kernel is as follows: firstly, each node in the brain network is labeled as their original ROI label. Then, the label of node is updated according to its previous label and the label of its neighboring node, and this process is repeated until the number of iterations reaches a predefined maximum value h. Finally, the WL subtree kernel on two graphs G and H with h iterations are defined as Equation (2) (Shervashidze et al., 2011):

(2) k(G,H)=< φ(G),φ(H)> 

where,

$$\begin{array}{l} \varphi \left(\text{G}\right)=(\sigma_{0}\left(\text{G},{\text{s}}_{01}\right),\cdots ,\sigma_{0}\left(\text{G},{\text{s}}_{0\left|{\text{L}}_{0}\right|}\right),\cdots ,\sigma_{\text{h}}\left(\text{G},{\text{s}}_{\text{h1}}\right),\cdots ,\\ \;\sigma_{\text{h}}\left(\text{G},{\text{s}}_{\text{h}\left|{\text{L}}_{\text{h}}\right|}\right)) \end{array}$$

and

$$\begin{array}{l} \varphi \left(\text{H}\right)=(\sigma_{0}\left(\text{H},{\text{s}}_{01}\right),\cdots ,\sigma_{0}\left(\text{H},{\text{s}}_{0\left|{\text{L}}_{0}\right|}\right),\cdots ,\sigma_{\text{h}}\left(\text{H},{\text{s}}_{\text{h1}}\right),\cdots ,\\ \;\sigma_{\text{h}}\left(\text{H},{\text{s}}_{\text{h}\left|{\text{L}}_{\text{h}}\right|}\right)) \end{array}$$

with σ_*i*_(G, s_*i, j*_) and σ_*i*_(H, s_*i, j*_) are the numbers of occurrences of the label s_*i, j*_ in G and H, respectively. s_*i, j*_ denotes the label of *i*-th node in iteration j. And |L_*i*_ | is the number of labels in iteration *i*. L_*i*_ denotes the set of labels of G and H in iteration *i*. L_0_ denotes the set of the initial labels of G and H. K is the kernel matrix of n × n and n is the number of brain networks.

After computing the graph kernel matrix, kernel PCA is performed to extract features. The kernel PCA is given by Equation (3):

(3) $$\lambda \alpha =\text{K}\alpha ,\;\alpha ={\left(\alpha_{1},\alpha_{2},\cdots ,\alpha_{\text{N}}\right)}^{\text{T}}$$

where λ and α are the eigenvalue and corresponding eigenvector of K. N is the number of networks. K is the kernel matrix computed using the WL subtree kernel.

Let $\alpha^{1},\alpha^{2},\cdots \;,\alpha^{\text{m}}\left(\alpha^{\text{m}}={\left\{\alpha_{1}^{\text{m}},\alpha_{2}^{\text{m}},\cdots \;,\alpha_{\text{N}}^{\text{m}}\right\}}^{\text{T}}\right)$ is the normalized eigenvector of the top-m biggest eigenvalues in Equation (3). Then, ${\text{U}}^{\text{m}}=\overset{\text{n}}{\underset{\text{i }=\;1}{\sum }}\alpha_{\text{i}}^{\text{m}}\varphi \left({\text{G}}_{\text{i}}\right)$ is the solution of Equation (3).

Therefore, for a network G, the new feature vector can be computed by Equation (4):

(4) $$\begin{array}{l} \begin{array}{l} ({\text{U}}^{\text{m}})\text{T}\cdot \varphi (\text{G})=\sum_{\text{i}=1}^{\text{n}}{\text{α}}_{\text{i}}^{\text{m}}\varphi^{\text{T}}({\text{G}}_{\text{i}})\cdot \varphi (\text{G})\\ \;=\sum_{\text{i}=1}^{\text{n}}{\text{α}}_{\text{i}}^{\text{m}}\text{K}({\text{G}}_{\text{i}},\text{G}) \end{array} \end{array}$$

**Figure 2:**
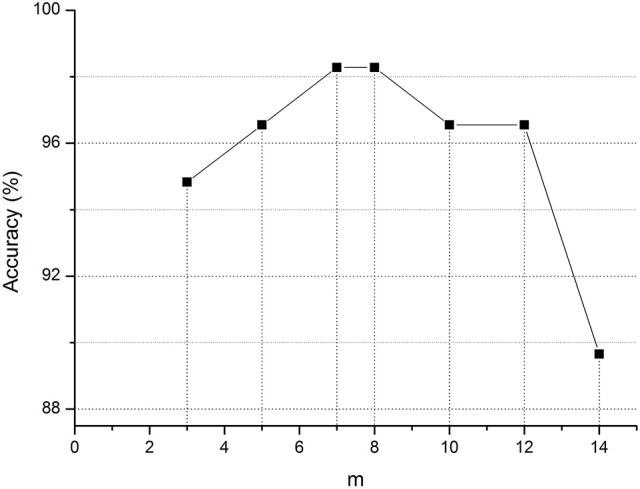
Classification accuracy of MCI for different m value. m represents the top-m biggest eigenvalues in graph kernel PCA.

Moreover, in graph kernel PCA, we simply use the top-m biggest eigenvalues. To evaluate the effect of m, we perform a list of MCI classification tasks with different values, the results of which are shown in Figure 2. This figure clearly shows that for *m* = 7 or 8, the accuracy is the highest in the classification of MCI and NC. At this point, m just satisfies the formula $\overset{\text{m}}{\underset{\text{i }=\;1}{\sum }}|\lambda_{\text{i}}|>0.9\times \overset{\text{n}}{\underset{\text{i }=\;1}{\sum }}|\lambda_{\text{i}}|$ (where *n* is the number of networks). It contains enough discriminative information. Therefore, in graph kernel PCA, we simply use the top-m biggest eigenvalues so that $\overset{\text{m}}{\underset{\text{i }=\;1}{\sum }}|\lambda_{\text{i}}|>0.9\times \overset{\text{n}}{\underset{\text{i }=\;1}{\sum }}|\lambda_{\text{i}}|$.

#### SVM and cross-validation

Finally, a simple SVM classifier (Chang and Lin, 2011) was adopted to identify AD patients and MCI patients from NCs. To evaluate the performance of this method, a 10-fold cross-validation (Chang and Lin, 2011) is adopted. Specifically, the subject dataset was randomly divided into 10 parts, one of which was left as the testing set, while the remaining nine were used as training sets. In this study, 10-fold cross-validation was performed 100 times to obtain more accurate results. Finally, we computed the arithmetic mean of the 100 repetitions as the final result.

### Implementation details

In our study, The MST brain network containing 90 nodes is constructed by the MST method based on the fully connected network obtained by preprocessing. In gSpan, the support value s is set as 0.7 to mine the frequency subnetwork in the MST brain network. The most discriminative subnetworks are selected from frequency subnetworks, and the brain network is rebuilt according to the most discriminative subnetworks. In the WL subtree kernel, the parameters h and n are set as 5 and 1, respectively. In the kernel PCA, the parameter target_dim m is set as 8, 20, and 18 for MCI vs. NC, AD vs. NC, and AD vs. MCI, respectively.

In our experiments, the classification performance of different methods was evaluated using accuracy, sensitivity, specificity and area under receiver operating characteristic (ROC) curve (AUC). Specifically, the accuracy measures the proportion of subjects that are correctly predicted among all subjects, the sensitivity represents the proportion of positives that are predicted correctly, and the specificity denotes the proportion of negatives that are predicted correctly. The ROC curve is a graphical plot that shows the diagnostic ability of a binary classifier system. It is created by plotting the sensitivity against 1-specificity over all possible values of the marker.

## Results

### Classification results

**Figure 3:**
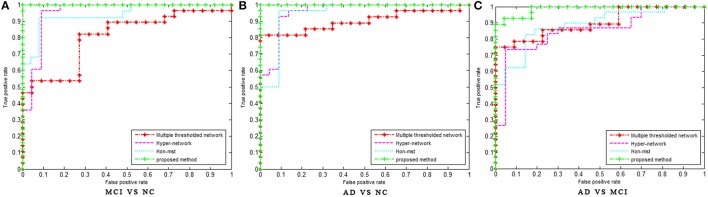
The ROC curve of different methods. The ROC curve of different methods on MCI vs. NC **(A)**, AD vs. NC **(B)**, and AD vs. MCI **(C)** classification. Multiple threshold, Multiple thresholded functional brain network; Hyper-network, Hyper-connectivity of functional brain networks; Hon-mst, Minimum spanning tree high-order functional brain network.

In this experiment, the MST was constructed, and frequent subnetwork was defined as the feature, while graph kernel PCA was used for feature selection. For classification of AD and MCI, the accuracy was 77.3%, the specificity was 100% and the sensitivity was 54.1%, AUC was 0.97. For classification of MCI and NC, the accuracy was 98.3%, the specificity was 100% and the sensitivity was 96.6%, AUC was 0.99. For classification of AD and NC, the accuracy was 91.3%, the specificity was 100% and the sensitivity was 82.5%, AUC was 1 (see Table 2). Figure 3 shows the ROC curve of the proposed method. The results showed that the classification method with subnetwork selection and graph kernel principal component analysis based on MST brain functional network could accurately distinguish AD, MCI, and NC subjects.

**Table 2:** Comparison of classification performance from different methods.

**Method**	**Task**	**ACC (%)**	**SEN (%)**	**SPE (%)**	**AUC**
Jie et al., 2014b	MCI/NC	91.9	100	88	0.94
Jie et al., 2016	MCI/NC	94.6	91.7	96.0	0.96
Guo et al., 2017a	AD/NC	98.2	98.9	96.7	0.998
Proposed method	MCI/NC	98.3	96.6	100	0.99
	AD/NC	91.3	82.5	100	1
	AD/MCI	77.3	54.1	100	0.97

*AD, Alzheimer's disease; NC, Normal control; MCI, Mild Cognitive Impairment; ACC, Accuracy; SEN, Sensitivity; SPE, Specificity; AUC, The area under the receiver operating characteristic curve*.

### Most discriminative subnetworks

**Figure 4:**
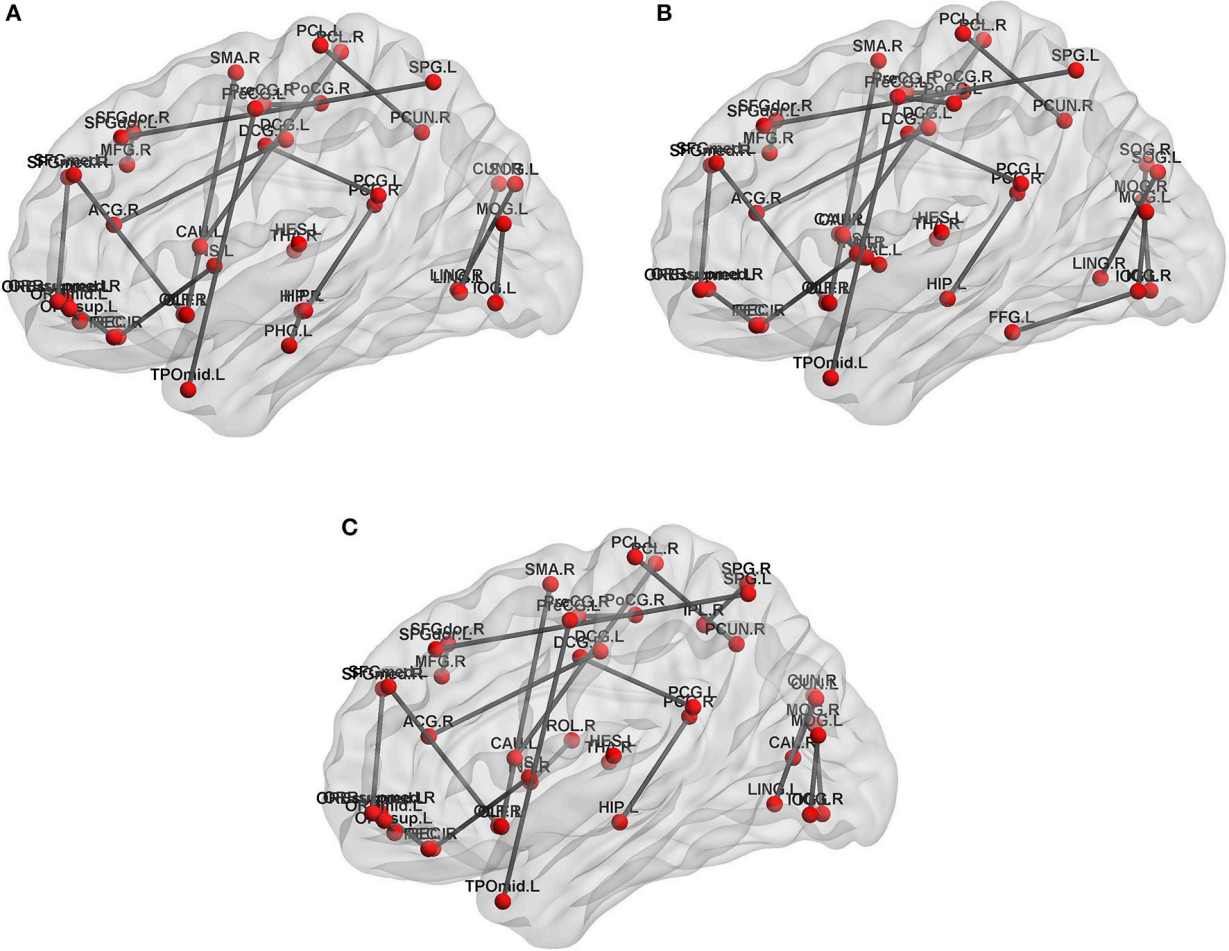
Frequent subnetworks of NC, AD and MCI. Frequent subnetwork mined by gSpan for MCI **(A)**, AD **(B)**, and NC **(C)** groups. PreCG.L, L Precental gyrus; PreCG.R, R Precental gyrus; SFGdor.L, L Superior frontal gyrus, dorsolateral; SFGdor.R, R Superior frontal gyrus, dorsolateral; ORBsup.L, L Superior frontal gyrus, orbital part; MFG.R, R Middle frontal gyrus; ORBmid.L, L Middle frontal gyrus, orbital part; ROL.R, R Rolandic operculum; SMA.R, R Supplementary motor area; OLF.L, L Olfactory cortex; OLF.R, R Olfactory cortex; SFGmed.L, L Superior frontal gyrus, medial; SFGmed.R, R Superior frontal gyrus, medial; ORBsupmed.L, L Superior frontal gyrus, medial orbital; ORBsupmed.R, R Superior frontal gyrus, medial orbital; REC.L, L Gyrus rectus; REC.R, R Gyrus rectus; INS.L, L Insula; INS.R, R Insula; ACG.R, R Anterior cingulate and paracingulate gyri; DCG.L, L Median cingulate and paracingulate gyri; DCG.R, R Median cingulate and paracingulate gyri; PCG.L, L Posterior cingulate gyrus; PCG.R, R Posterior cingulate gyrus; HIP.L, L Hippocampus; HIP.R, R Hippocampus; PHG.L, L Parahippocampal gyrus; CAL.R, R Calcarine fissure and surrounding cortex; CUN.L, L Cuneus; CUN.R, R Cuneus; LING.L, L Lingual gyrus; LING.R, R Lingual gyrus; SOG.L, L Superior occipital gyrus; SOG.R, R Superior occipital gyrus; MOG.L, L Middle occipital gyrus; MOG.R, R Middle occipital gyrus; IOG.L, L Inferior occipital gyrus; IOG.R, R Inferior occipital gyrus; FFG.L, L Fusiform gyrus; PoCG.L, L Postcentral gyrus; PoCG.R, R Postcentral gyrus; SPG.L, L Superior parietal gyrus; SPG.R, R Superior parietal gyrus; IPL.R, R Inferior parietal, but supramarginal and angular gyri; PCUN.R, R Precuneus; PCL.L, L Paracentral lobule; PCL.R, R Paracentral lobule; CAU.L, L Caudate nucleus; CAU.R, R Caudate nucleus; PUT.L, L Lenticular nucleus, putamen; PUT.R, R Lenticular nucleus, putamen; PAL.L, L Lenticular nucleus, pallidum; THA.R, R Thalamus; HES.L, L Heschl gyrus; TPOmid.L, L Temporal pole: middle temporal gyrus.

In feature extract, frequent subnetwork were mined from MST brain network by gSpan (support is set as 0.7). Figure 4 depicts frequent subnetwork of MCI, AD, and NC.

**Figure 5:**
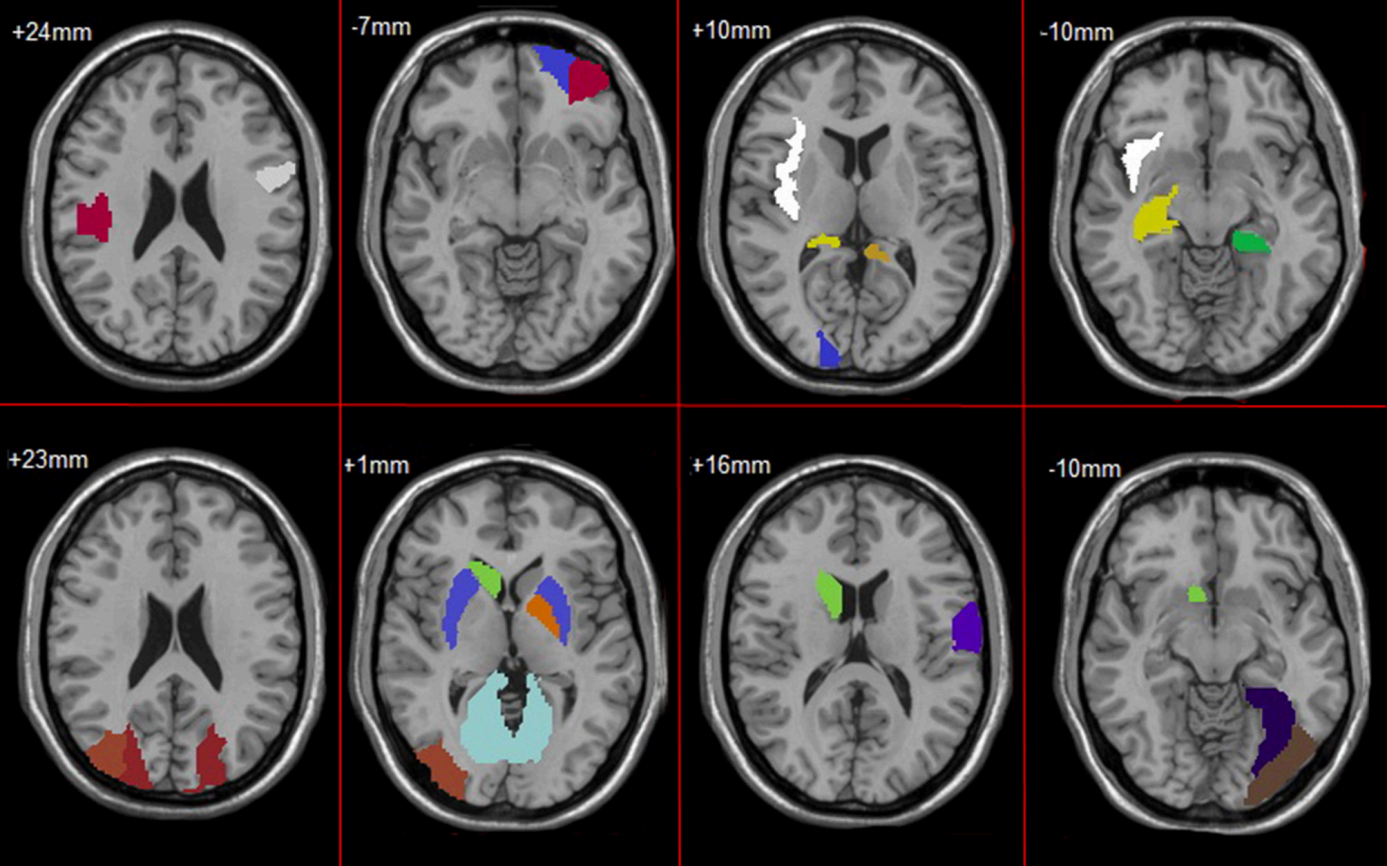
Most discriminative regions that were selected using the proposed method in AD.

**Figure 6:**
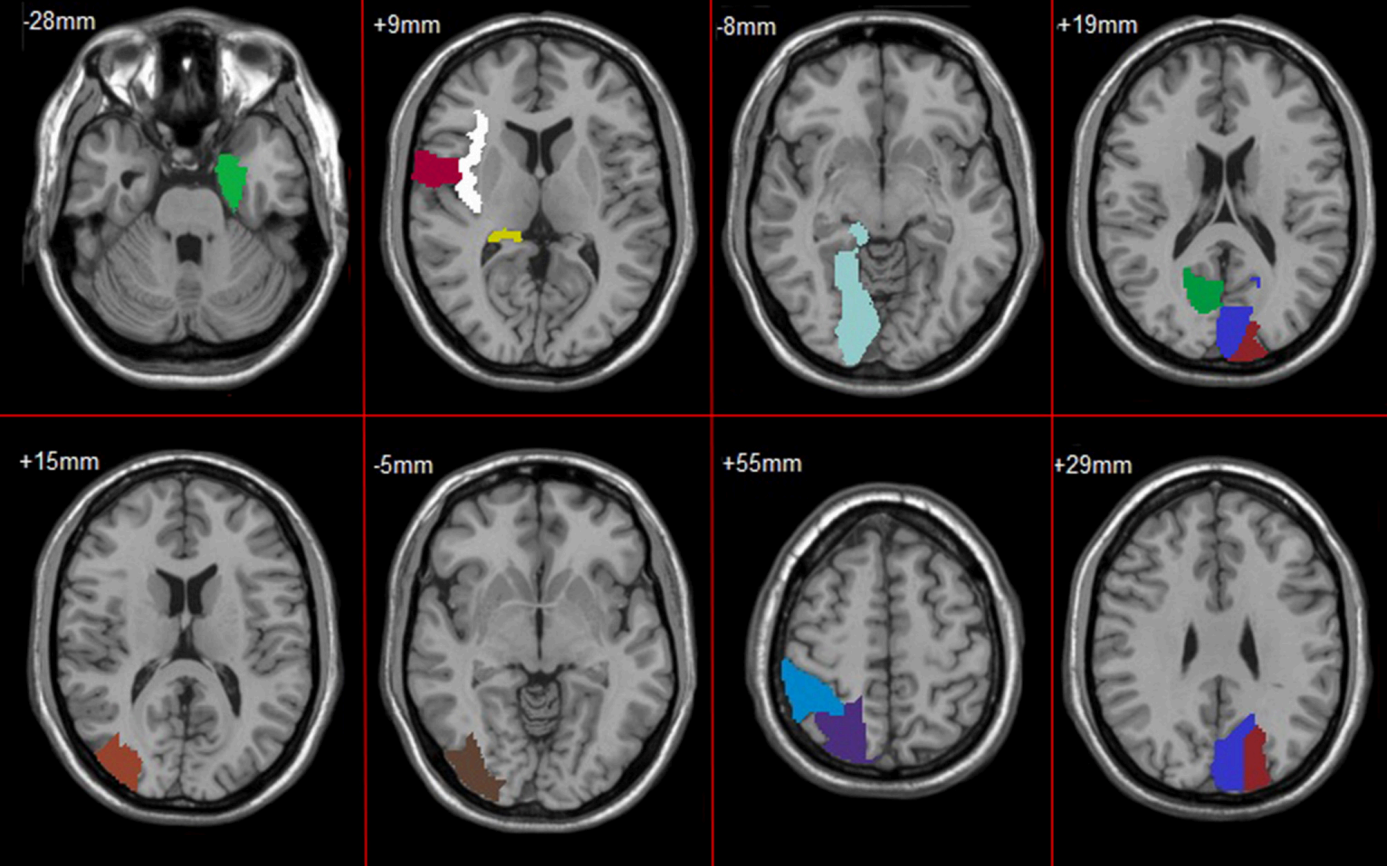
Most discriminative regions that were selected using the proposed method in MCI.

In feature selection, we choose those subnetworks with the highest frequency difference as the most discriminative subnetworks for classification. Figures 5, 6 show most discriminative regions.

The result shows that the most discriminative subnetworks obtained using our proposed method include the precentral gyrus, orbitofrontal cortex, insula, superior occipital gyrus, hippocampus, and fusiform gyrus; these findings are consistent with those of previous studies.

## Discussion

In this article, we proposed a classification framework based on MST brain functional networks to automatically identify AD patients, MCI patients, and NC. This framework used MST to construct a brain network, gSpan to mine frequent subnetworks, and graph kernel PCA to select the most discriminative subnetwork for classification. The classification performance was evaluated by using 10-fold cross-validation. The experimental results show that our proposed method can achieve significantly improved classification performance compared to other state-of-the-art methods.

### Classification performance

The human brain is a very complex system. Brain diseases cause a series of changes in the brain. Recently, connectivity-network-based classification methods have been proposed for the diagnosis of AD, MCI, and NC. In the conventional classification methods, the researchers extracted features from the threshold network and formed long vector to train a classifier for classification. For example, the clustering coefficient was extracted as feature for MCI classification (Wee et al., 2011). The local network measures were extracted as feature for MCI classification (Wee et al., 2012a,b). The weights between the regions of interest pairs were extracted for classification (Chen et al., 2011). However, one disadvantage of those methods is that some useful network topological information was not fully considered, which limits the further improvement of classification performance. Jie et al. (2014b) used the topological information to identify MCI patients. Jie et al. (2014a) proposed a framework to integrate network topological and connectivity properties for improving the classification performance. However, in the construction of a threshold network, threshold setting will affect the performance of the classification to a certain extent.

In our study, brain functional network is constructed by using an MST brain network and topological information are extracted from MST networks for final classification. We compared our method with existing methods. Table 2 shows the classification performances of different methods. As can be seen from the Table 2, the proposed method has the best classification accuracy, sensitivity, specificity, and AUC value, especially in the classification of MCI and NC. Specifically, for classification of MCI and NC, the proposed method achieves classification accuracy of 98.3%, sensitivity of 96.6%, specificity of 100%, and AUC of 0.99; in comparison, for other classification methods, the best accuracy is only 94.6%, best sensitivity is 100%, best specificity is 96%, and best AUC is 0.96. However, we also observed the low sensitivity of the proposed method for AD/MCI classification. There may be two reasons for this. Firstly, MCI was considered a transitional stage between NC and early AD. MCI is a stage of progressive global cognitive decline, including the loss of memory, reasoning, and language. According to a study by Liu et al. (2018), abnormalities in functional integrity and functional compensation coexist in patients with MCI, so the difference between AD and MCI was not obvious. Secondly, in our experiment, the number of most discriminative regions between AD and MCI was 17, but 13 of them also appeared in AD and NC, and only 4 of them appeared in MCI and NC. That is to say, most discriminative regions between AD and MCI covered the typical damaged brain regions in AD patients. So the classifier can accurately identify typical AD patients without misdiagnosis. However, due to the existence of compensation mechanism, the degree of brain damage in some AD patients was not significant, resulting in the missed diagnosis of some AD patients.

In order to avoid the influence of different data sets on the results, the network construction methods and classification features of the existing studies (Jie et al., 2014b, 2016; Guo et al., 2017a) were used in the experiment, and the classification performance was compared with the same data sets. Table 3 shows the classification performances of different methods with same dataset. Figure 3 shows the ROC curve of these different methods. The result showed that the proposed method is superior to the other three methods, especially in MCI/NC classification, which indicates that this method can capture the early features of disease. In addition, the specificity of proposed method is 100%, which indicates that the method can accurately identify the patients without misdiagnosis. In addition, compared with the proposed methods, these methods were more complex in network construction and feature selection. Specifically, in Jie et al's. (2014b) method, it was necessary to construct several functional connectivity networks with different thresholds, and extract topological properties of the network as features to classify. In Jie et al.'s (2016) method, hyper-networks was constructed by sparse representation, and three different types of clustering coefficients was used as feature to classify. In Guo et al.'s (2017a) method, the low-order and high-order networks must be constructed firstly, then the MST high-order functional connectivity network can be constructed. In addition, appropriate threshold need to be set for feature selection. The selection of threshold will affect the performance of classification. These factors increase the complexity of the methods.

**Table 3:** Classification performance of different methods with the same dataset.

**Method**	**Research**	**Task**	**ACC (%)**	**SEN (%)**	**SPE (%)**	**AUC**
Multiple threshold	Jie et al., 2014b	MCI/NC	75.7	74.3	78.1	0.81
		AD/NC	78.5	73.3	85.6	0.9
		AD/MCI	74	90	42.2	0.9
Hyper-network	Jie et al., 2016	MCI/NC	80.8	76.7	80	0.94
		AD/NC	88.3	91.7	86.7	0.95
		AD/MCI	77.5	60	95	0.85
Hon-mst	Guo et al., 2017a	MCI/NC	82.6	84.1	87.5	0.93
		AD/NC	94.2	95.1	95.4	0.95
		AD/MCI	80.7	73.3	85	0.89
MST	Proposed method	MCI/NC	98.3	96.6	100	0.99
		AD/NC	91.3	82.5	100	1
		AD/MCI	77.3	54.1	100	0.97

*Multiple threshold, Multiple thresholded functional brain network; Hyper-network, Hyper-connectivity of functional brain networks; Hon-mst, Minimum spanning tree high-order functional brain network; MST, Minimum spanning tree functional brain network; AD, Alzheimer's disease; NC, Normal control; MCI, Mild cognitive impairment; ACC, Accuracy; SEN, Sensitivity; SPE, Specificity; AUC, The area under the receiver operating characteristic curve*.

In conclusion, the results showed that the simple, unbiased brain network constructed by MST and the topological properties of the network captured by graph kernel PCA can improve the classification performance. Therefore, our proposed method can achieve better results while using the unbiased brain networks and fewer features.

### Discriminative brain regions

The regions selected in the course of classification by our method are in agreement with previous studies and include the precentral gyrus (Lenzi et al., 2011), orbitofrontal cortex (Wee et al., 2012b), insula (Wee et al., 2012b), posterior cingulate cortex (Zhang et al., 2007, 2010), superior occipital gyrus (Wee et al., 2012b), Fusiform gyrus (Whitwell et al., 2007), hippocampus (Shen et al., 2010), and parahippocampal gyrus (Wee et al., 2012b), Putamen of lenticular nucleus (de Jong et al., 2008).

Specifically, the orbitofrontal cortex participates in the cognitive process of the brain during decision-making and reflects emotion and reward in the decision-making (Supekar et al., 2008). If the prefrontal cortex is damaged, it will affect the brain's control of emotion and mood. The posterior cingulate cortex is the hub node in the default mode network and participates in various functions of the brain network. It plays a prominent role in pain and memory (Nielsen et al., 2005). The precentral gyrus involved in the transfer of attention and eye movement (Lenzi et al., 2011). The insula are believed to be involved in consciousness and play an important role in perception, motor control, self-awareness, language, cognitive functioning, emotions, and interpersonal experience. Fusiform gyrus has been linked with various neural pathways related to recognition. The hippocampus plays important roles in spatial memory and in the consolidation of information from short-term memory to long-term memory. The hippocampus demonstrated a significantly negative correlation to episodic memory performance (Bai et al., 2009). The Parahippocampal gyrus plays an important role in the encoding and recognition of environmental scenes (Machulda et al., 2001). The main function of the putamen is to regulate movements and influence learning.

Additionally, the other observation is that the hippocampus, parahippocampal gyrus, and insula are the first regions of the brain to suffer damage. It is agree with the fact that Alzheimer's disease is always forgetting recent events and decline in attention, language, and executive in early stage. As the disease advances, some regions were damaged, such as L Orbital part of superior frontal gyrus, L Orbital part of middle frontal gyrus, Posterior cingulate cortex, L Fusiform gyrus, L Postcentral gyrus and Putamen. This loss results in some problems with disorientation, cognitive decline, mood swings, incapable of self-care, loss of motivation, and behavioral problems. And, these regions were parts of default mode network (DMN). It proves that the dysfunction of DMN is closely related to AD.

### Effect of MST

To investigate the effect of MST on the classification performance, we performed the same experiment on threshold networks and MST networks. A study has shown (Zanin et al., 2012) that a brain function network with sparsity of 40% demonstrated higher classification performance. Therefore, a brain function network with the sparsity of 40% was constructed for comparison. Specifically, a functional full connected network obtained by preprocessing can be represented as the correlation matrix. The threshold is set according to the sparsity (40%) of the network, and then the correlation matrix of the fully connected network is transformed into binary matrix according to the threshold. That is, if the weight is greater than the threshold, the corresponding element of the binary matrix is 1, otherwise it is 0. Thus, a brain function network with the sparsity of 40% was constructed. Finally, the discriminative subnetwork selection method and graph kernel PCA method were used to extract features, a linear SVM was trained for classification. Table 4 shows the classification performances.

**Table 4:** Classification performance of threshold-based and MST-based methods.

**Method**	**Task**	**ACC (%)**	**SEN (%)**	**SPE (%)**	**AUC**
Threshold-based	MCI/NC	63.3	73.3	65	0.65
	AD/NC	87.5	85	76.7	0.92
	AD/MCI	65.8	66.7	81.7	0.76
Proposed method	MCI/NC	98.3	96.6	100	0.99
	AD/NC	91.3	82.5	100	1
	AD/MCI	77.3	54.1	100	0.97

*Threshold-based denotes a brain function network with the sparsity of 40%; AD, Alzheimer's disease; NC, Normal control; MCI, Mild cognitive impairment; ACC, Accuracy; SEN, Sensitivity; SPE, Specificity; AUC, The area under the receiver operating characteristic curve*.

Obviously, these results indicated that the choice of threshold affects the structure and properties of the network, and affects the performance of the classification to a certain extent. The uniqueness of MST facilitates a comparison between brain networks. This conclusion is consistent with previous results (Tewarie et al., 2015).

### Effect of KPCA

To evaluate the effect of feature extraction based on graph kernel PCA, we directly use discriminative subnetworks as features for classification. Specifically, let *f*_*ij*_ denote the jth feature of the network *G*_*i*_. If the jth discriminative subnetwork is a subnetwork of the network *G*_*i*_, then *f*_*ij*_ is 1, otherwise it is 0. Accordingly, we can obtain feature vectors for every brain network. Then, we use SVM for classification. Table 5 summarizes the classification performances.

**Table 5:** Classification performance when directly using discriminative subnetworks and that of our proposed method.

**Method**	**Task**	**ACC (%)**	**SEN (%)**	**SPE (%)**	**AUC**
Discriminative subnetwork	MCI/NC	78.3	66.7	80	0.78
	AD/NC	88.2	82.3	94.1	0.97
	AD/MCI	69.1	71.2	67.4	0.76
Proposed method	MCI/NC	98.3	96.6	100	0.99
	AD/NC	91.3	82.5	100	1
	AD/MCI	77.3	54.1	100	0.97

*Discriminative subnetwork denotes directly use discriminative subnetworks as features; AD, Alzheimer's disease; NC, Normal control; MCI, Mild cognitive impairment; ACC, Accuracy; SEN, Sensitivity; SPE, Specificity; AUC, The area under the receiver operating characteristic curve*.

As shown in Table 5, the results of our proposed method are better than those of the method in which discriminative subnetworks are directly used as features in terms of accuracy, sensitivity, specificity, and AUC. These results show that feature extraction based on graph kernel PCA plays an important role in our proposed method. This is because graph kernel PCA can not only measure the similarity between two brain networks by comparing the topological structure of the network, but also can map the feature data from high dimension to low dimension, so as to cover most of the data information with very few features. More recently, some researchers have used graph kernels in neuroimaging studies. For example, Du et al. (2016) used graph kernel PCA to select features for classification of Attention Deficit Hyperactivity Disorder (ADHD) patients.

### Limitations

Through the experimental analysis, our method has obtained higher classification accuracy and specificity, but the sensitivity needs to be improved. In the actual diagnosis, the doctor should combine the image data with the result of neuropsychological questionnaires to make a diagnosis. Therefore, the combination of image data and data of neuropsychological questionnaires may further improve the performance of the classification, which will be explored in the future. In addition, because of the small amount of data used in the experiment, the results of the classification are lack of generality. This method is applied to larger AD dataset in future work.

## Conclusion

In this paper, we have proposed an MST classification framework to identify AD patients, MCI patients, and NCs. The proposed method mainly used the MST method, gSpan, and graph kernel PCA. Specifically, MST was used to construct the brain functional connectivity network; gSpan, to extract features; and graph kernel PCA, to select features.

In experiments with the ADNI dataset, our proposed method not only can significantly improve classification performance in terms of accuracy, sensitivity, specificity, and AUC value, but also can potentially detect the ROIs that are sensitive to disease pathology. In future work, we will explore the combination of image data and data of neuropsychological questionnaires.

## Ethics statement

This study was approved by the medical ethics committee of Shanxi Province, and the approved certification number is 2012013. All subjects have been given written informed consent in accordance with the Declaration of Helsinki.

## Author contributions

XC proposed a minimum spanning tree classification framework. GY, HZ, and FL processed data and made experiment. JX, HG, XC, and JC gave the proof of results. All the authors have read through the manuscript and approved it for publication. JC had full access to all of the data in the study and takes responsibility for its integrity and the accuracy of data analysis.

### Conflict of interest statement

The authors declare that the research was conducted in the absence of any commercial or financial relationships that could be construed as a potential conflict of interest. The reviewer XG and handling Editor declared their shared affiliation.
